# Sulfonamide‐Bearing Pyrazolone Derivatives as Multitarget Therapeutic Agents: Design, Synthesis, Characterization, Biological Evaluation, In Silico ADME/T Profiling and Molecular Docking Study

**DOI:** 10.1002/prp2.70088

**Published:** 2025-03-24

**Authors:** Nebih Lolak, Suleyman Akocak, Meryem Topal, Ümit Muhammet Koçyiğit, Mesut Işık, Cüneyt Türkeş, Fevzi Topal, Mustafa Durgun, Şükrü Beydemir

**Affiliations:** ^1^ Department of Pharmaceutical Chemistry Faculty of Pharmacy, Adıyaman University Adıyaman Turkey; ^2^ Vocational School of Health Services Gümüşhane University Gümüşhane Turkey; ^3^ Department of Biochemistry Faculty of Pharmacy, Sivas Cumhuriyet University Sivas Turkey; ^4^ Department of Bioengineering Faculty of Engineering, Bilecik Şeyh Edebali University Bilecik Turkey; ^5^ Department of Biochemistry Faculty of Pharmacy, Erzincan Binali Yıldırım University Erzincan Turkey; ^6^ Department of Food Engineering Faculty of Engineering and Natural Sciences, Gümüşhane University Gümüşhane Turkey; ^7^ Department of Chemical and Chemical Processing Technologies, Laboratory Technology Program Gümüşhane University Gümüşhane Turkey; ^8^ Department of Chemistry Faculty of Arts and Sciences, Harran University Şanlıurfa Turkey; ^9^ Department of Biochemistry Faculty of Pharmacy, Anadolu University Eskişehir Turkey

**Keywords:** carbonic anhydrase inhibitors, cholinesterases inhibitors, glaucoma, molecular docking, sulfonamide‐bearing pyrazolones

## Abstract

The research and design of new inhibitors for the treatment of diseases such as Alzheimer's disease and glaucoma through inhibition of cholinesterases (ChEs; acetylcholinesterase, AChE and butyrylcholinesterase, BChE) and carbonic anhydrase enzymes are among the important targets. Here, a series of novel sulfonamide‐bearing pyrazolone derivatives (**1a–f** and **2a–f**) were successfully synthesized and characterized by using spectroscopic and analytical methods. The inhibitory activities of these newly synthesized compounds were evaluated both in vitro and *in silico* for their effect on carbonic anhydrases (*h*CA I and *h*CA II isoenzymes) and ChEs. The in vitro studies showed that these novel compounds demonstrated potential inhibitory activity, with *K*_I_ values covering the following ranges: 18.03 ± 2.86–75.54 ± 4.91 nM for *h*CA I, 24.84 ± 1.57–85.42 ± 6.60 nM for *h*CA II, 7.45 ± 0.98–16.04 ± 1.60 nM for AChE, and 34.78 ± 5.88–135.70 ± 17.39 nM for BChE. Additionally, many of these compounds showed promising inhibitory activity, and some showed higher potency than reference compounds. While the *in silico* studies have also identified the potential binding positions of these compounds, using the crystal structures of *h*CA I, II, AChE and BChE receptors. The varying affinities demonstrated by these designed compounds for ChEs and *h*CA isoenzymes show that these compounds could hold promise as potential alternative agents for selectively inhibiting ChEs and *h*CAs in the treatment of diseases such as Alzheimer's disease and glaucoma.

## 1 Introduction

Carbonic anhydrases (CAs; EC 4.2.1.1) are recognized for their pivotal role in maintaining acid–base balance in body fluids and tissues by catalyzing the rapid conversion of water and carbon dioxide (CO_2_) into bicarbonate (HCO_3_^−^) and protons (H^+^). The kidneys and lungs are the most important tissues organs involved in this buffering system in metabolic processes. Fifteen CA isoenzymes have been identified from seven genetically distinct families, denoted as α‐, β‐, γ‐, δ‐, ζ‐, η‐, and θ‐CA [1, 2, 3, 4]. These isozymes exhibit varying catalytic activities and inhibitory affinities in bodily fluids and tissues. Among them, cytosolic CA I and CA II are extensively researched isoforms [5, 6]. CA I is associated with conditions like cerebral edema and retinal diseases, while CA II is linked to disorders such as glaucoma, epilepsy, and edema [7, 8]. The inhibitory effects of substances/drugs on CA isozymes can vary [9]. Reports suggest that CA isozyme inhibitors find potential applications in the treatment of diseases such as leukemia, diabetes mellitus (DM), cystic fibrosis, epilepsy, and neurological disorders. The pathophysiological mechanisms of diabetes mellitus (DM) could potentially be associated with the regulation of carbonic anhydrase (CA) activity in diabetes [10, 11]. Many studies have reported the potential activity of alcohols, sulfonamides, phenol acetate, and alkyl or aryl carboxylic acids as CA inhibitors [12]. Sulfonamides are recognized for their high affinity toward CAs due to their interaction with the zinc binding group (ZBG) in the active site of CA isozymes [13, 14]. Primary sulfonamides and their isosteres (sulfamates, sulfamides, etc.) stand out as highly effective ZBG for the purpose of designing inhibitors targeting the metallo‐enzymes carbonic anhydrases (CA, EC 4.2.1.1). In recent years, there has been a notable emergence of novel carbonic anhydrase inhibitors (CAIs) based on sulfonamide moieties [15, 16, 17, 18, 19].

Many drugs and agents currently in clinical development, which are classified as sulfonamide or sulfamate derivatives, have demonstrated significant carbonic anhydrase inhibition activity. Beyond their traditional applications as diuretics and anti‐glaucoma agents, recent findings have revealed their potential roles as anticonvulsants, anti‐obesity drugs, anti‐cancer agents, pain relievers, and anti‐infective medications [20]. However, critical challenges in designing CAIs as therapeutic agents are associated with the high number of isoforms found in humans. Their widespread distribution in various tissues and organs can result in a lack of isoform selectivity for the currently available sulfonamide/sulfamate‐type inhibitors. Inhibitory profiles against the 15 mammalian isoenzymes can vary significantly among compounds that selectively inhibit certain CA isoforms with therapeutic value, offering opportunities for structure‐based drug design of next‐generation, isoform‐selective inhibitors [21]. Pharmaceuticals in clinical use, owing to their potential activity against other *h*CA isoenzymes or their effects on metabolic pathways, may induce certain side effects. Consequently, there is a need to design and develop novel agents with varying levels of selectivity for *h*CA isoenzymes to mitigate these adverse effects [22].

Alzheimer's disease (ad) stands out as one of the most prevalent forms of dementia, affecting individuals aged 65 and older. Besides the neuropathological markers of ad, it is characterized by a consistent deficit in cholinergic neurotransmission, particularly affecting cholinergic neurons in the basal forebrain [23]. In the cholinergic system, choline acetyltransferase (ChAT) is involved in acetylcholine synthesis, while cholinesterases (acetylcholinesterase EC 3.1.1.7; AChE) and butyrylcholinesterase (EC 3.1.1.8; BChE) play a role in its degradation [24]. These cholinesterase enzymes can disrupt neural transmission by breaking down the neurotransmitter acetylcholine in the synaptic cleft. Notably, AChE exhibits greater specificity for the neurotransmitter ACh compared to butyrylcholinesterase (BChE) [25]. Individuals with ad exhibit known reductions in brain ACh levels. To counteract the decrease in neurotransmitter levels hydrolyzed by cholinesterases, a widely adopted approach has been the use of acetylcholinesterase inhibitors (AChEIs). One of the earliest drugs used for this purpose was 1,2,3,4‐tetrahydro‐9‐aminoacridine (tacrine), specifically approved for ad treatment in 1993 [26]. Currently, various AChEIs, including donepezil, galantamine, and rivastigmine, are available for the symptomatic treatment of mild‐to‐moderate ad. Cholinesterase inhibitory therapy may be viewed as a straightforward, symptomatic, short‐term intervention by its pharmacological nature. Some clinical studies indicate that the beneficial effects can be extended for up to 36 months [27]. However, it is important to note that these medications carry the potential for serious side effects such as vasodilation, bradycardia, weight loss, bronchoconstriction, and miosis [28].

**FIGURE 1:**
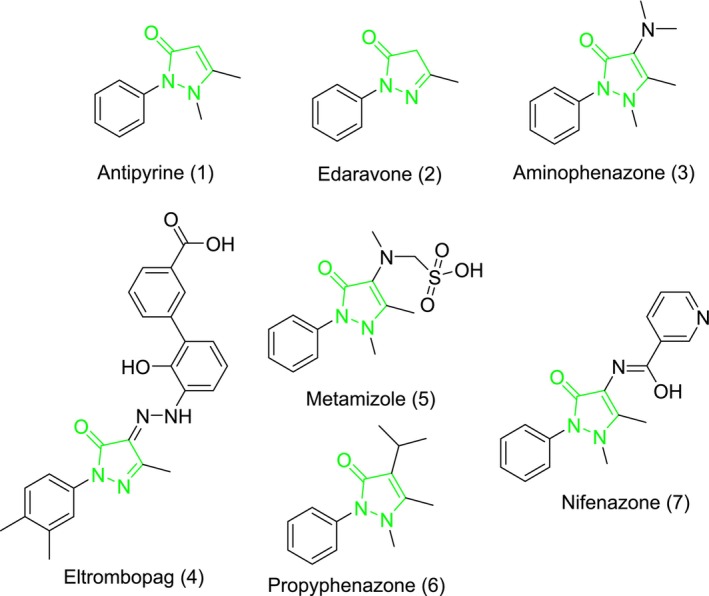
FDA has approved drugs with a pyrazolone nucleus as well as investigational pyrazolone derivatives.

Pyrazolones are a widely studied heterocyclic compound due to their many properties and applications. This compound consists of a five‐membered lactam ring with two nitrogen atoms and one ketonic group within its structure. The uses of pyrazolones include non‐narcotic analgesics, antipyretics, anti‐inflammatory agents, and treatment for rheumatoid arthritis [29, 30]. They are also used in the synthesis of dyes (they can undergo various chemical reactions to produce chromophoric compounds, which are essential for imparting color to textiles, inks, and paints) and agrochemicals (these compounds can stimulate or inhibit the growth of plants, influence their flowering and fruiting processes, and enhance crop yields). The most well‐known pyrazolone is antipyrine [29], which was used as a painkiller and antipyretic agent in the past. However, due to its side effects, it is no longer used in clinical practice. At present, multiple FDA‐approved drugs containing the pyrazolone nucleus have been explored (Figure 1), including edaravone [30], which is used as a free radical scavenger for the treatment of amyotrophic lateral sclerosis (ALS) [31], and aminophenazone [29], which has antipyretic and anti‐inflammatory properties and is used in breath tests to measure cytochrome P‐450 metabolic activity in liver function evaluations. Other drugs featuring the pyrazolone moiety encompass eltrombopag [31], metamizole [32], propyphenazone [33], and nifenazone [34] (Figure 1). Therefore, the development and synthesis of novel compounds containing the pyrazolone moiety as potential metabolic enzyme inhibitors have garnered significant interest among researchers. In this study, a series of sulfonamide‐bearing pyrazolone derivatives (**1a–f** and **2a–f**) were synthesized and comprehensively characterized using various spectroscopic and analytical techniques. Subsequently, the inhibitory activities of these synthesized compounds against ChEs and *h*CA isoenzymes were determined through in vitro and *in silico* studies. Furthermore, molecular docking analysis was conducted to understand the binding interactions of the most effective inhibitor with the active sites of ChEs and *h*CA isoenzymes, shedding light on their inhibitory activities.

### 1.1 Result and Discussions

### 1.2 Chemistry

**SCHEME 1:**
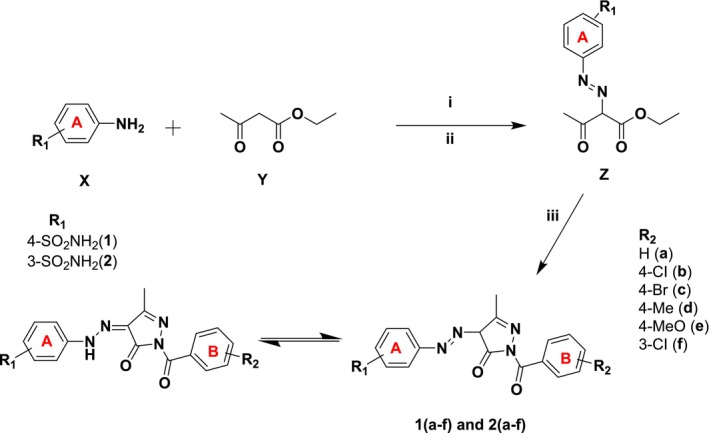
General synthetic pathway for the synthesis of 1‐substitutedbenzoyl‐3‐methyl‐5‐oxo‐4,5‐dihydro‐1H‐pyrazol‐4‐yl diazenyl benzenesulfonamide derivatives (**1a‐f** and **2a‐f**). Reagents and conditions: (i) NaNO_2_/HCl, H_2_O, 0°C–5°C; (ii) Ethylacetoacetate, MeOH, sodium acetate, 0°C–10°C; (iii) Hydrazide derivatives (R_2_CONHNH_2_), EtOH, piperidine, reflux, 8–10 h.

**FIGURE 2:**
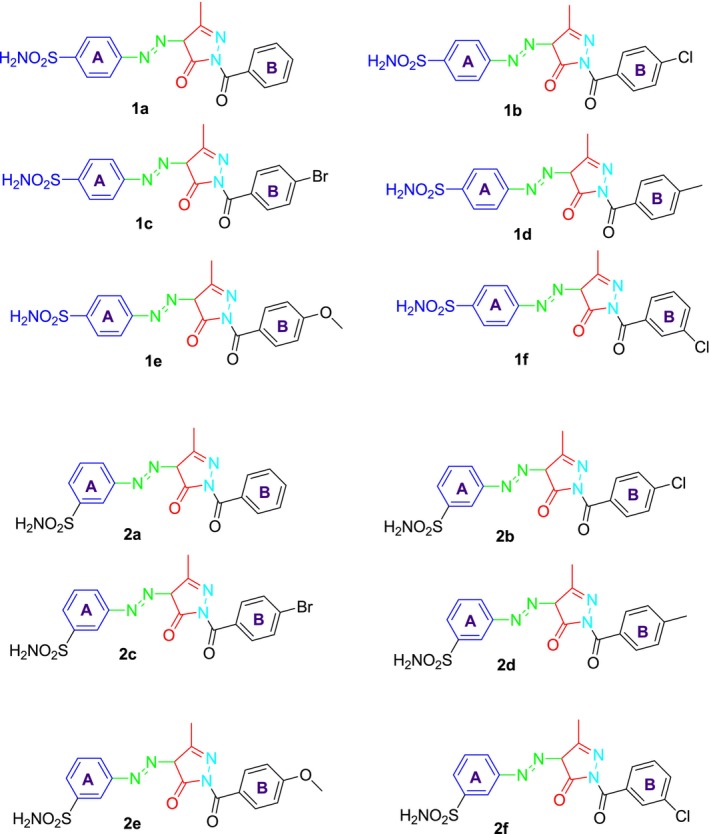
Structure of novel pyrazolones bearing benzenesulfonamide derivatives synthesized as potential carbonic anhydrase and cholinesterase inhibitors (**1a–f** and **2a–f**).

Pyrazoles are widely recognized as aromatic heterocycles featuring two nitrogen atoms within their five‐membered rings. This versatile heterocyclic family encompasses a wide array of synthetic and natural compounds renowned for their diverse chemical, biological, agrochemical, and pharmacological properties [35]. The condensation of 1,3‐diketones, β‐ketoesters, and 2,4‐diketoesters with hydrazine or hydrazide derivatives has been extensively utilized in synthesizing pyrazole derivatives [36]. In this research, the synthesis of the sulfonamide‐bearing pyrazolone derivatives was accomplished in three steps following a literature protocol [37]. In the first step, sulfanilamide or metanilamide **1** was diazotized and then treated in the second step at the same temperature with ethyl acetoacetate to give the diazo‐containing ethyl acetoacetate intermediate **2**, which was then treated in the final step with various arylhydrazides using piperidine as a catalyst in refluxing EtOH to give the target sulfonamide‐bearing pyrazolone derivatives in excellent yields (78%–95%). To obtain these molecules in their pure form, they underwent comprehensive purification using the crystallization method, followed by the determination of their melting points. Subsequently, their chemical structures were elucidated through ^1^H and ^13^C‐NMR, as well as FT‐IR spectroscopic analyses. The reaction sequences employed for the synthesis of the target sulfonamide‐bearing pyrazolone derivatives are illustrated in Scheme 1. The chemical structure of the synthesized compounds is presented in Figure 2.

### 1.3 Biological Evaluation

Pyrazole derivatives stand out as an particularly active group of compounds, with a broad spectrum of biological activities such as anti‐inflammatory, antiviral, antitumor, antidepressant, and antioxidant activities [38, 39, 40]. In recent years, the pharmaceutical industry has harnessed the potential of pyrazole derivatives, resulting in the development of several drugs [38, 39, 40]. The prominence of the pyrazole ring in numerous pharmaceutically active compounds can be attributed to its straightforward synthesis and pharmacological efficacy. Moreover, the selective functionalization of pyrazole with various substituents has further expanded its utility across various domains [35, 41, 42, 43]. These individual and distinct features of the pyrazole ring have directed attention toward the design of alternative pyrazole derivatives that have varying selectivity toward ChEs and *h*CA isoenzymes. In the existing literature, several studies have delved into the synthesis of pyrazole derivatives and their impact on ChEs and *h*CAs [38, 39, 40]. Notably, a recent investigation unveiled novel pyrazole derivatives, affirming their effective inhibition against AChE enzymes and cytosolic *h*CA I and II [38, 39, 40]. These pyrazole derivatives exhibited inhibition with *K*_I_ values ranging from 48.94 ± 9.63 to 116.05 ± 14.95 μM for AChE, 1.03 ± 0.23 to 22.65 ± 5.36 μM for *h*CA I, and 1.82 ± 0.30 to 27.94 ± 4.74 μM for *h*CA II [44]. Another study introduced a series of novel pyrazole derivatives with effective inhibition profiles, featuring *K*_I_ values spanning from 5.13 to 67.39 nM against *h*CA I and II [45]. Furthermore, researchers explored pyrazole‐based carbohydrazone hybrids, revealing synthesized derivatives with varying inhibitory potential, characterized by *K*_I_ values within the range of 572.8–10 000 nM for *h*CA I, 6.8–10 000 nM for *h*CA II, and 10.1–10 000 nM for *h*CA IX [46]. Additionally, novel sulfamides incorporating the dopamine scaffold were synthesized, leading to the inhibition of carbonic anhydrases across all CA isozymes. The *K*_I_ values for CA I ranged from 0.061 to 1.822 μM, while for CA II, they were in the nanomolar range, between 1.47 and 2.94 nM [47]. In the context of Alzheimer's disease (ad), a distinct series of thiazole‐piperazine hybrids has recently received wide interest in this field as these compounds exhibited notable inhibitory potential against AChE, with *IC*_50_ values calculated as 0.0496 ± 0.002 μM, 0.0317 ± 0.001 μM, and 0.2158 ± 0.010 μM for compounds **3a**, **3c**, and **3i**, respectively [48].

In this study, a series of novel sulfonamide‐bearing pyrazolone derivatives (**1a–f** and **2a–f**) were synthesized and their inhibitory effects on metabolic enzymes were investigated in vitro against ChEs and *h*CA isoenzymes. Table 1 provides a summary of the results that correspond to the given information. The synthesized derivatives displayed substantial inhibitory potency against both ChEs and *h*CAs, engaging in competitive and non‐competitive inhibition mechanisms at the nanomolar (nM) scale. *K*_I_ values for *h*CA I encompassed a range of 18.03 ± 2.86–75.54 ± 4.91 nM, whereas for *h*CA II, they spanned from 24.84 ± 1.57 to 85.42 ± 6.60 nM. Noteworthy is the fact that the newly developed derivatives exhibited superior inhibitory efficacy when compared to the reference compound AAZ (*K*_I_ of 382.13 ± 17.26 nM for *h*CA I and *K*_I_ of 87.48 ± 3.62 nM for *h*CA II) against *h*CA isoenzymes. Among the synthesized compounds, compound **2d** emerged as the most potent inhibitor against *h*CA I, displaying a remarkable *K*_I_ value of 18.03 ± 2.86 nM, acting in a competitive manner. Conversely, compound **1e** exhibited the lowest inhibitory efficacy among the derivatives against *h*CA I, with a *K*_I_ value of 75.54 ± 4.91 nM, also functioning as a non‐competitive inhibitor. Upon examining the *K*_I_ values, which are known indicators of the enzyme's affinity for the inhibitor and its selectivity, it is generally observed that compound **1e** exhibits the lowest selectivity for *h*CA isozymes, while **2d** showed the highest selectivity. Regarding *h*CA II, compound **2b** exhibited non‐competitive inhibitory effects with *K*_I_ values in the range of 24.84 ± 1.57 nM, whereas compound **1c** displayed comparatively weaker potency, with a *K*_I_ value of 85.42 ± 6.60 nM, also acting as a non‐competitive inhibitor (Table 1). Compound **2b**, apart from compound **2d**, demonstrated greater selectivity for *h*CA I in comparison to the other compounds. Moreover, it showed increased selectivity for *h*CA II compared to all other compounds.

**TABLE 1:** Inhibition data of important metabolic enzymes with the novel sulfonamide‐bearing pyrazolone derivatives (**1a–f** and **2a–f**) and reference inhibitors, acetazolamide and tacrine.

Compound ID	*h*CA I^a^		*h*CA II^b^		AChE^c^		BChE^d^	
	*K*_I_ (nM)^e^	*R* ^2^ ^f^	*K*_I_ (nM)^e^	*R* ^2^ ^f^	*K*_I_ (nM)^e^	*R* ^2^ ^f^	*K*_I_ (nM)^e^	*R* ^2^ ^f^
1a	53.88 ± 5.03	0.9603	65.55 ± 5.42	0.9634	11.20 ± 1.15	0.9916	95.54 ± 5.78	0.9851
1b	55.86 ± 3.29	0.9816	50.76 ± 3.36	0.9812	13.32 ± 1.43	0.9864	73.33 ± 12.24	0.9602
1c	49.22 ± 4.27	0.9608	85.42 ± 6.60	0.9662	9.47 ± 1.23	0.9845	34.78 ± 5.88	0.9682
1d	65.05 ± 3.00	0.9894	42.63 ± 2.92	0.9782	16.04 ± 1.60	0.9875	64.58 ± 7.59	0.9809
1e	75.54 ± 4.91	0.9770	63.70 ± 6.71	0.9835	13.99 ± 1.49	0.9870	80.90 ± 9.41	0.9791
1f	66.81 ± 5.15	0.9706	62.25 ± 4.19	0.9812	7.45 ± 0.98	0.9901	58.20 ± 8.11	0.9706
2a	73.21 ± 7.17	0.9522	55.73 ± 4.14	0.9787	10.53 ± 0.90	0.9939	98.30 ± 7.34	0.9766
2b	37.06 ± 2.43	0.9765	24.84 ± 1.57	0.9846	8.66 ± 0.93	0.9902	76.43 ± 11.62	0.9666
2c	44.57 ± 2.84	0.9791	28.84 ± 2.51	0.9633	8.43 ± 1.42	0.9759	96.03 ± 5.44	0.9882
2d	18.03 ± 2.86	0.9688	36.16 ± 2.78	0.9703	15.77 ± 1.04	0.9945	88.57 ± 12.92	0.9673
2e	48.99 ± 6.25	0.9438	35.43 ± 1.94	0.9869	13.50 ± 0.91	0.9948	93.85 ± 10.47	0.9816
2f	37.67 ± 2.06	0.9840	33.28 ± 4.09	0.9224	9.52 ± 0.86	0.9940	135.70 ± 17.39	0.9765
AAZ^g^	382.13 ± 17.26	0.9872	87.48 ± 3.62	0.983	—	—	—	—
THA^h^	—	—	—	—	108.03 ± 11.82	0.9832	156.72 ± 13.62	0.9815

^a^ Human carbonic anhydrase I.

^b^ Human carbonic anhydrase II.

^c^ Acetylcholinesterase.

^d^ Butyrylcholinesterase.

^e^ Inhibition constant.

^f^ Coefficient of determination of *K*_I_ value.

^g^ Acetazolamide.

^h^ Tacrine.

In cholinesterase assays, the synthesized compounds exhibited inhibitor potential with *K*_I_ values ranging from 7.45 ± 0.98 to 16.04 ± 1.60 nM for AChE and from 34.78 ± 5.88 to 135.70 ± 17.39 nM for BChE. Notably, all the tested derivatives showed higher inhibition against ChEs than the reference compound THA (*K*_I_ of 108.03 ± 11.82 nM for AChE and *K*_I_ of 156.72 ± 13.62 nM for BChE). Among these compounds, compound **1f** displayed very strong inhibition activity against the BChE enzyme with a remarkable *K*_I_ value of 7.45 ± 0.98 nM, acting in a competitive manner. Conversely, compound **1d** showed lower inhibition activity than others with a *K*_I_ value of 16.04 ± 1.60 nM, also functioning as a competitive inhibitor. Regarding BChE, compound **1c** exhibited substantial inhibitory potential, boasting a *K*_I_ value of 34.78 ± 5.88 nM while operating as a competitive inhibitor, whereas compound **2f** displayed comparatively weaker potency than others, with a *K*_I_ value of 135.70 ± 17.39 nM, also acting as a competitive inhibitor (Table 1). Compound **2f** shows higher selectivity for AChE rather than BChE. Also, compound **1f** displayed higher selectivity for BChE compared to other compounds except compound **1c**. Moreover, it exhibited higher selectivity for AChE compared to all other compounds.

Upon meticulous analysis of the results, it is clear that all compounds exhibit inhibitory properties against both ChEs and *h*CA isozymes. Significantly, compounds **2b** and **2d** emerge as prominent compounds within this group, demonstrating heightened selectivity for *h*CA isoenzymes. Furthermore, all compounds display potent inhibitory effects on ChEs and show a greater selectivity toward AChE over BChE.

Through the investigation of structure–activity relationships, it was possible to discern the influence of the sulfonamide group's position on the benzene ring **A** on the inhibitory activity against the targeted carbonic anhydrase isozymes. It was observed that all compounds with a sulfonamide group at the meta position displayed higher activity than their counterparts with a sulfonamide group at the para position, with the exception of compounds **1a** and **2a**. In these exceptions, the position of the sulfonamide group did not seem to have the same effect on activity, potentially due to the absence of electron‐withdrawing substituents on the benzene ring **B**, a feature that is present in all other compounds. Furthermore, the evaluation of sulfonamide‐bearing pyrazolone derivatives against cholinesterases (acetylcholinesterase AChE and butyrylcholinesterase BChE) showed that the position of the sulfonamide substituent on the benzene ring **A** of these compounds (meta‐vs. para‐) was correlated to selectivity for activity against these two enzymes. This relatively small change in structure had a substantial effect on activity. Specifically, all compounds bearing the meta sulfonamide group exhibited higher activity against the acetylcholinesterase enzyme compared to their counterparts linked to the sulfonamide group in the para position, with the exception of compounds **1f** and **2f**, which are distinguished from the other derivatives by having an electron‐withdrawing group **Cl** attached to the meta position instead of the para position, as is the case with all other derivatives. The meta chloro compound **1f** (*K*_I_ value 7.45 ± 0.98 nM) that possesses a para sulfonamide moiety at an aromatic ring **A** was the most potent derivative and exhibited stronger AChE inhibitory activity than the reference Tacrine compound (*K*_*I*_ value 7.45 ± 0.98 nM). In relation to the butyrylcholinesterase BChE enzyme, compounds containing a paraulfonamide moiety at ring **A** were more active than corresponding compounds containing a metaulfonamide structure.

The inhibition data acquired from this study underscore the superior effectiveness of the synthesized derivatives in comparison to reference compounds. Furthermore, these derivatives demonstrate comparable or even heightened inhibitory effects when compared to numerous compounds documented in the existing literature. These substantial inhibitory effects not only enhance the potential of novel pyrazole derivatives as promising candidates for further exploration in the field of enzyme inhibition but also emphasize the significance of structural modifications in the development of new compounds for potential application in the treatment of glaucoma and ad.

### 1.4 Molecular Docking Studies

**FIGURE 3:**
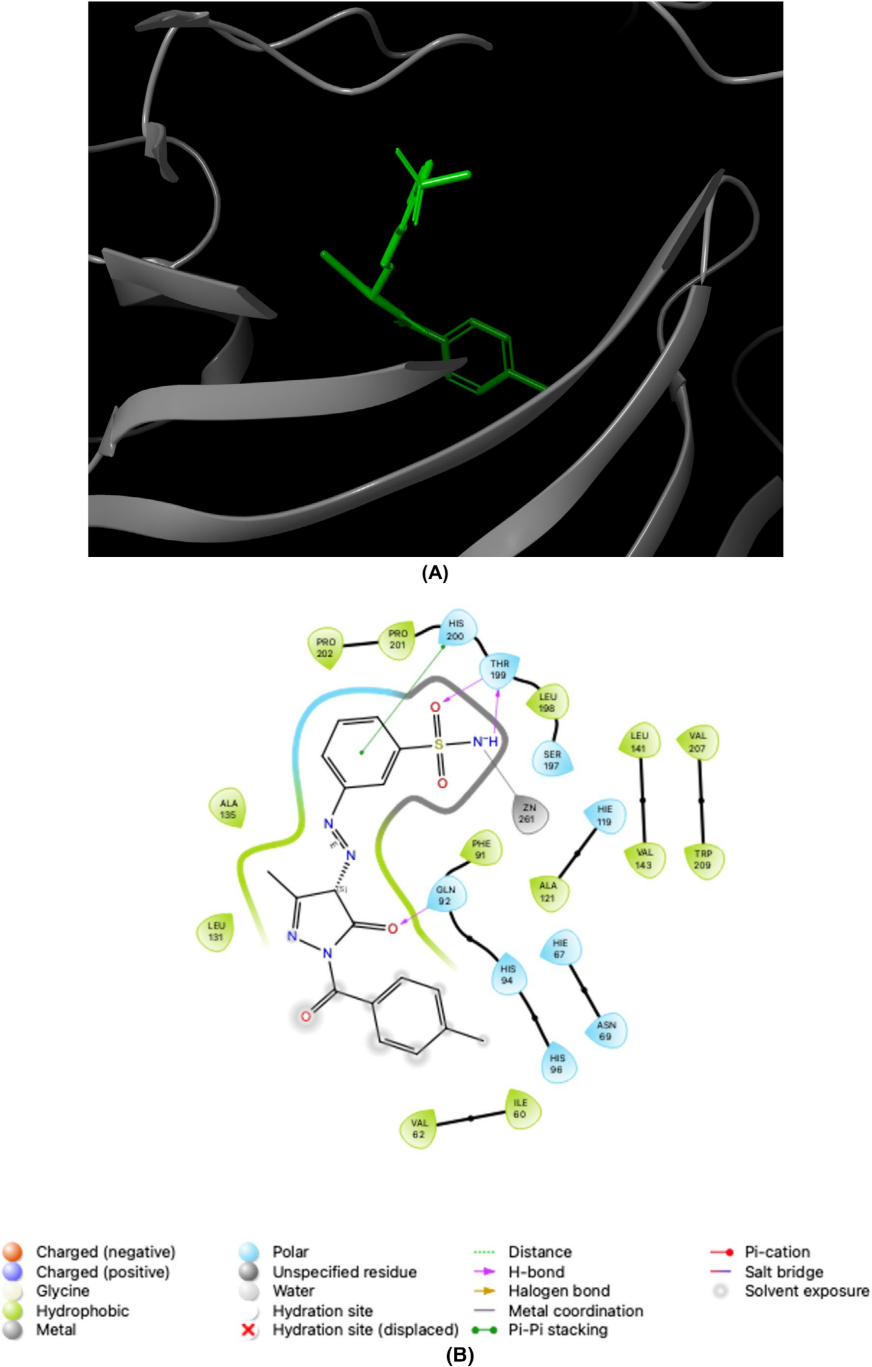
Illustrates the application of molecular docking to the *h*CA I, which corresponds to the PDB ID 1AZM. The purpose of this process was to dock the 1AZM with 3‐([3‐Methyl‐1‐(4‐methylbenzoyl)‐5‐oxo‐4,5‐dihydro‐1*H*‐pyrazol‐4‐yl]diazenyl) benzenesulfonamide (referred to as **2d**). As a result, the compound **2d** was successfully docked within the binding pocket of 1AZM, as depicted in (A). Additionally, (B) presents a 2D interaction diagrams that elucidate the specific interactions between 1AZM and compound **2d**.

**FIGURE 4:**
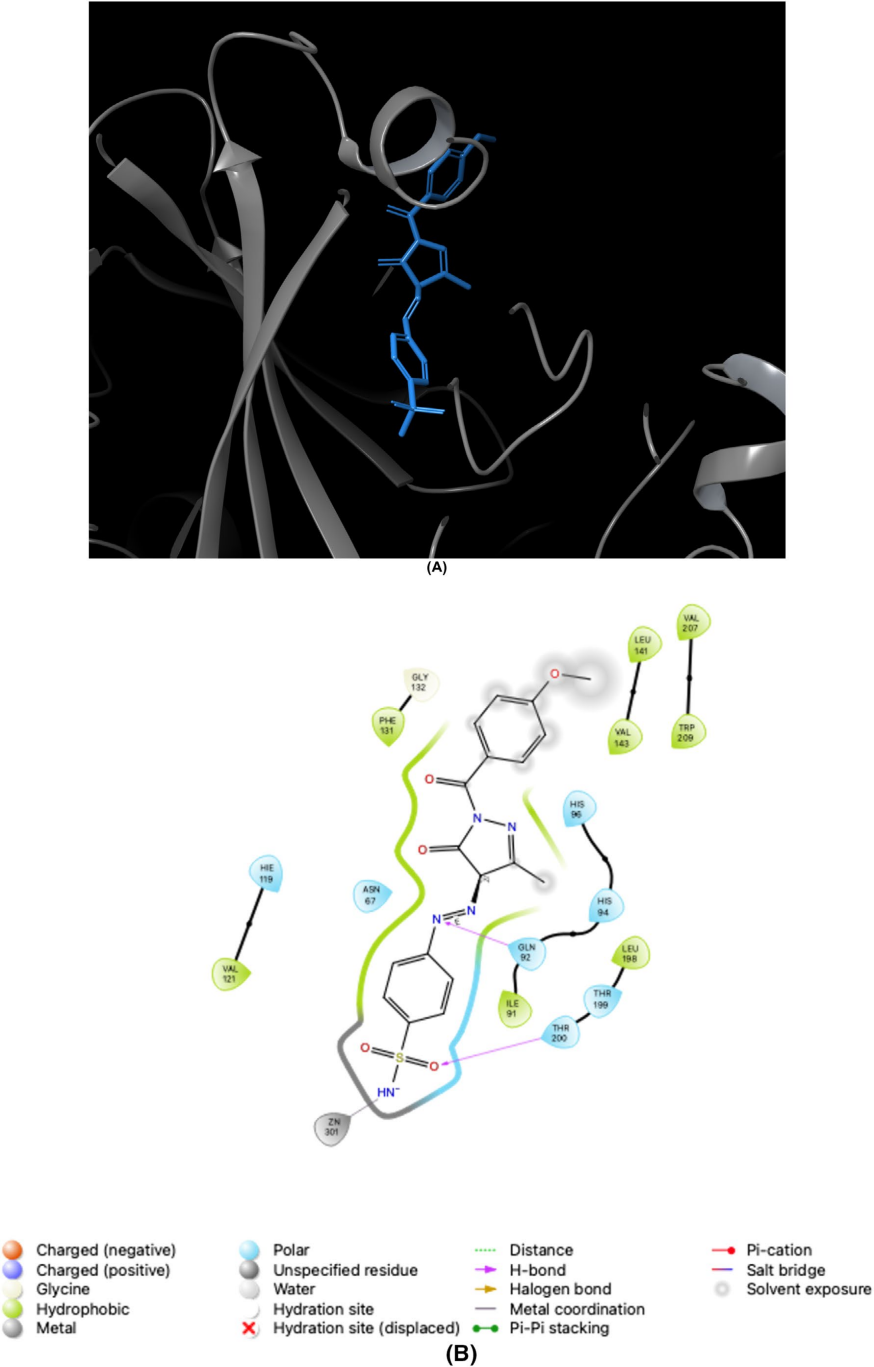
Illustrates the application of molecular docking to the *h*CA II, which corresponds to the PDB ID 3HS4. The purpose of this process was to dock the 3HS4 with 4‐([1‐(4‐Methoxybenzoyl)‐3‐methyl‐5‐oxo‐4,5‐dihydro‐1*H*‐pyrazol‐4‐yl]diazenyl) benzenesulfonamide (referred to as **1e**). As a result, the compound **1e** was successfully docked within the binding pocket of 3HS4, as depicted in (A). Additionally, (B) presents a 2D interaction diagrams that elucidate the specific interactions between 3HS4 and compound **1e**.

**FIGURE 5:**
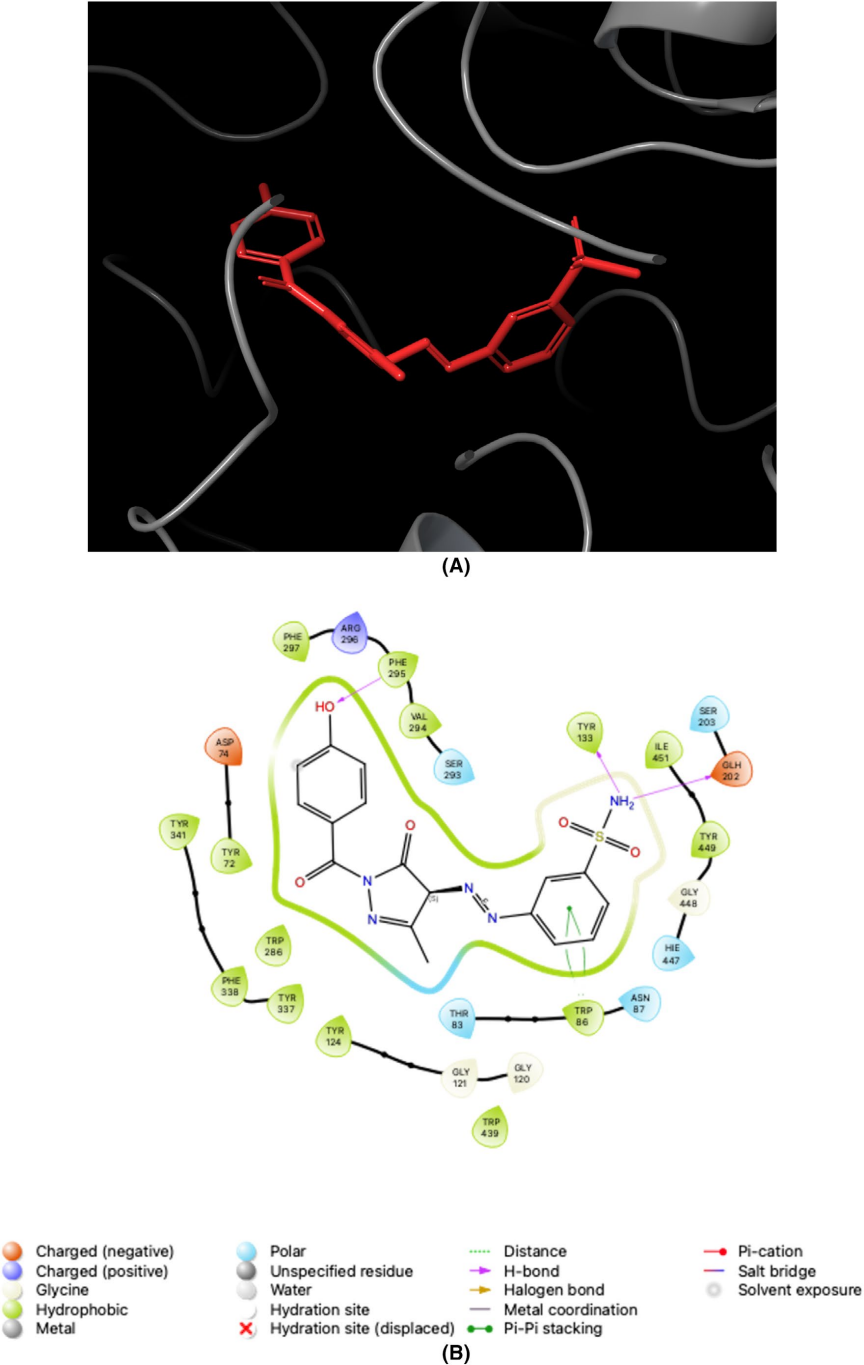
Illustrates the application of molecular docking to the AChE, which corresponds to the PDB ID 7XN1. The purpose of this process was to dock the 7XN1 with 4‐([1‐(3‐Chlorobenzoyl)‐3‐methyl‐5‐oxo‐4,5‐dihydro‐1*H*‐pyrazol‐4‐yl]diazenyl) benzenesulfonamide (referred to as **1f**). As a result, the compound **1f** was successfully docked within the binding pocket of 7XN1, as depicted in (A). Additionally, (B) presents a 2D interaction diagrams that elucidate the specific interactions between 7XN1 and compound **1 f**.

**FIGURE 6:**
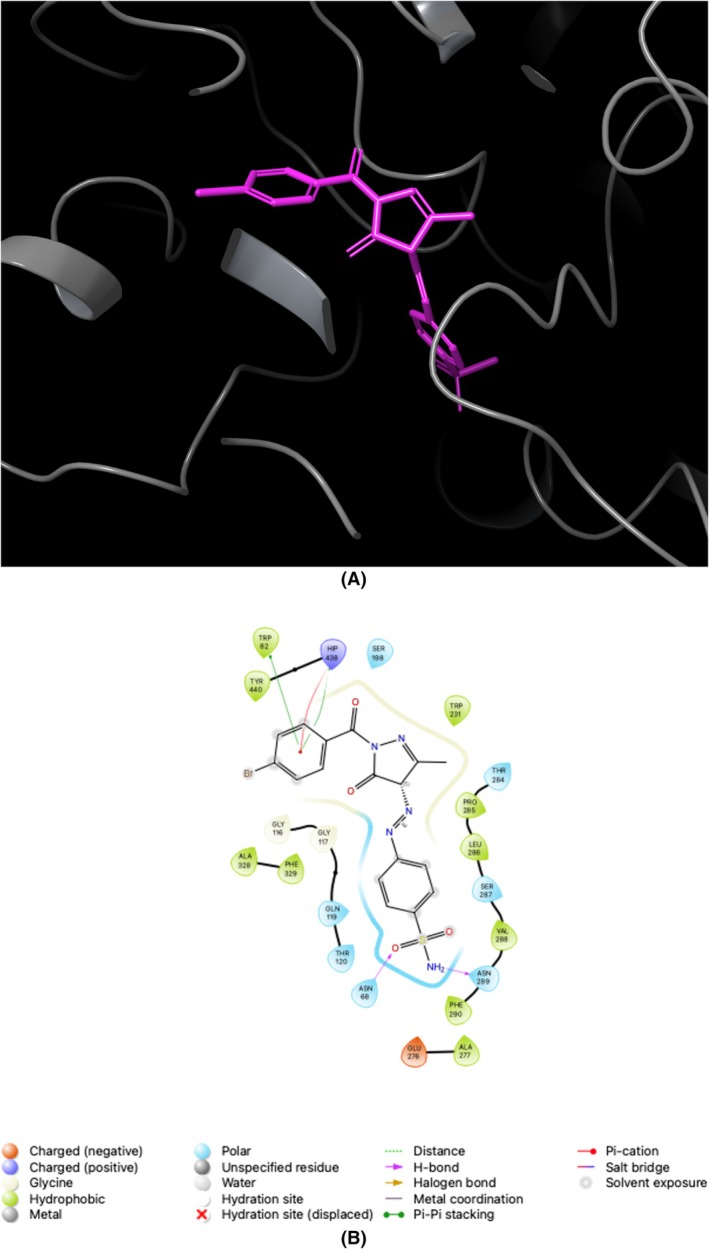
Illustrates the application of molecular docking to the BChE, which corresponds to the PDB ID 4BDS. The purpose of this process was to dock the 4BDS with 4‐([1‐(4‐Bromobenzoyl)‐3‐methyl‐5‐oxo‐4,5‐dihydro‐1*H*‐pyrazol‐4‐yl]diazenyl) benzenesulfonamide (referred to as **1c**). As a result, the compound **1c** was successfully docked within the binding pocket of 4BDS, as depicted in (A). Additionally, (B) presents a 2D interaction diagram that elucidates the specific interactions between 4BDS and compound **1c**.

The assessment of the efficacy of the Glide XP docking protocol was conducted by performing re‐docking of the co‐crystallized native ligands, specifically AAZ/AZM (5‐acetamido‐1,3,4‐thiadiazole‐2‐sulfonamide) for *h*CAs and THA (1,2,3,4‐tetrahydroacridin‐9‐amine) for ChEs, into the active sites of these enzymes (PDB IDs; 1AZM [49] and 3HS4 [50] for *h*CAs, and 7XN1 [51] and 4BDS [52] for ChEs) using Small‐Molecule Drug Discovery Suite 2023–4 for Mac. Determining the root mean square deviation values between the conformation of the native ligands (AAZ and THA) and the optimal pose generated by this protocol resulted in values of 0.15, 1.41, 0.04, and 0.03 Å for *h*CA I and II, AChE, and BChE, respectively. The findings mentioned above strongly suggested that the Glide XP docking algorithm is suitable for the docking process of novel sulfonamide‐bearing pyrazolone derivatives (**1a–f** and **2a–f**) to the active pockets of the *h*CAs and ChEs. Figures 3, 4, 5, 6 serve as prime examples that provide evidence for the fact that the docking scores of the most potent competitive inhibitors, **2d** (for *h*CA I), **1e** (for *h*CA II), **1f** (for AChE), and **1c** (for BChE), specifically −7.01 kcal/mol, −5.07 kcal/mol, −10.43 kcal/mol, and − 5.43 kcal/mol, respectively, demonstrate the existence of comparable conformations. It is worth noting that these conformations are characterized by various interactions, such as hydrogen bonding and π‐π stacking, which occur within the corresponding enzyme‐binding pockets. In other words, these conformations involve intricate molecular interactions between the ligands and the enzyme‐binding pockets, stabilizing the ligands within the pockets. Furthermore, an evaluation was conducted on the drug‐like characteristics of novel sulfonamide‐bearing pyrazolone derivatives (**1a–f** and **2a–f**) using the QikProp module, which is an integral component of the Schrödinger Suite 2023–4 designed for Mac operating systems. To assess their suitability as potential drugs, a comprehensive analysis was performed on a selection of ADME/T parameters, and the results have been meticulously computed and recorded in Table 2. The results of this study suggest that, according to their physicochemical characteristics, the compounds **1a–f** and **2a–f** meet the requirements for molecules with drug‐like properties, as defined by the guidelines established by Lipinski [53] and Jorgensen [54].

**TABLE 2:** ADME/T‐related parameters^a^ of novel sulfonamide‐bearing pyrazolone derivatives (**1a–f** and **2a–f**) and the reference inhibitors acetazolamide and tacrine.

Compounds ID	CNS	MW	Dipole	Volume	donorHB	accptHB	QPlogPoct	QPlogPw	QPlogPo/w	QPlogS	QPlogHERG	QPPCaco	QPlogBB	QPlogKp	Metab	QPlogKhsa	HOA	PSA	Rule of Five	Rule of Three
1a	–2	385.4	6.4	1159.8	2.0	11.0	23.1	17.9	0.6	−3.9	−6.4	47.4	−2.3	−4.3	3	−0.5	60.5	154.8	0	0
1b	−2	419.8	4.4	1204.1	2.0	11.0	23.5	17.6	1.1	−4.6	−6.3	46.7	−2.2	−4.5	3	−0.4	63.2	154.8	0	0
1c	−2	464.3	4.7	1213.1	2.0	11.0	23.7	17.6	1.2	−4.7	−6.4	46.8	−2.2	−4.5	3	−0.4	63.6	154.8	0	0
1d	−2	399.4	13.8	1220.7	2.0	11.0	25.3	17.6	0.9	−4.4	−6.4	47.4	−2.4	−4.5	4	−0.4	62.2	155.0	0	0
1e	−2	415.4	13.4	1235.9	2.0	11.0	25.5	18.1	0.7	−4.1	−6.4	48.3	−2.4	−4.4	4	−0.5	61.2	162.9	0	0
1f	−2	419.8	11.9	1203.9	2.0	11.0	24.9	17.6	1.1	−4.6	−6.3	47.5	−2.2	−4.5	3	−0.4	63.3	154.8	0	0
2a	−2	385.4	5.5	1162.5	2.0	11.0	23.0	17.9	0.6	−4.0	−6.5	47.5	−2.3	−4.3	3	−0.5	60.6	154.9	0	0
2b	−2	419.8	5.0	1206.8	2.0	11.0	23.6	17.6	1.1	−4.7	−6.4	47.4	−2.2	−4.5	3	−0.4	63.4	154.9	0	0
2c	−2	464.3	5.2	1218.3	2.0	11.0	23.8	17.6	1.2	−4.8	−6.4	47.2	−2.2	−4.5	3	−0.4	63.9	154.9	0	0
2d	−2	399.4	13.2	1224.7	2.0	11.0	25.2	17.6	0.9	−4.5	−6.4	48.1	−2.4	−4.5	4	−0.4	62.5	155.0	0	0
2e	−2	415.4	14.0	1239.7	2.0	11.8	25.7	18.1	0.7	−4.1	−6.3	47.5	−2.4	−4.4	4	−0.5	61.2	163.1	0	0
2f	−2	419.2	12.3	1204.1	2.0	11.0	25.0	17.6	1.1	−4.6	−6.4	48.3	−2.2	−4.5	3	−0.4	63.5	154.9	0	0
AAZ^b^	−2	222.2	10.7	640.3	3.0	9.0	17.6	15.2	−1.7	−1.6	−3.8	33.2	−1.8	−5.9	1	−0.9	43.8	134.9	0	0
THA^c^	1	198.3	4.5	707.7	1.5	2.0	10.7	6.3	2.5	−3.1	−4.1	2874.1	32	−1.8	3	66	100.0	34.5	0	0

^a^ QikProp tool properties and descriptors: CNS, Central nervous system activity (−2 inactive, +2 active); MW, molecular weight of the molecule (130.0–725.0); Dipole, computed dipole moment of the compound (1.0–12.5); Volume, total solvent‐accessible volume in cubic angstroms using a probe with a 1.4 Å radius (500.0–2000.0); donorHB, estimated number of hydrogen bonds that would be donated by the solute to water molecules in an aqueous solution (0.0–6.0); accptHB, estimated number of hydrogen bonds that would be accepted by the solute from water molecules in an aqueous solution (2.0–20.0); QPlogPoct, octanol/gas partition coefficient (8.0–35.0); QPlogPw, water/gas partition coefficient (4.0–45.0); QPlogPo/w, octanol/water partition coefficient (−2.0–6.5); QPlogS, aqueous solubility (−6.5–0.5); QPlogHERG, IC_50_ value for blockage of HERG K^+^ channels (concern below −5); QPPCaco, apparent Caco‐2 cell permeability in nm/s (< 25 poor, great > 500); QPlogBB, brain/blood partition coefficient (−3.0 to 1.2); QPlogKp, skin permeability (−8.0 to –1.0); Metab, number of likely metabolic reactions (1–8); QPlogKhsa, prediction of binding to human serum albumin (−1.5 to 1.5); HOA, human oral absorption (< 25% poor, high > 80%); PSA, van der Waals surface area of polar nitrogen and oxygen atoms (7.0–200.0); Rule of Five, number of violations of Lipinski's rule of five (max. 4); and Rule of Three, number of violations of Jorgensen's rule of three (max. 2).

^b^ Acetazolamide.

^c^ Tacrine.

## 2 Conclusion

In this study, a series of novel pyrazolone derivatives incorporating sulfonamide moieties (**1a–f** and **2a–f**) were synthesized, and their inhibitory effects on metabolic enzymes, specifically ChEs and *h*CA isoenzymes, were evaluated. The synthesized derivatives demonstrated potent inhibitory activity against both ChEs and *h*CAs. Notably, these newly developed derivatives exhibited superior inhibitory efficacy compared to the reference compounds AAZ and THA against *h*CAs and ChEs, respectively. Upon thorough analysis of the results of this study, it became apparent that variations in the R_1_ and R_2_ substituents within the pyrazolones bearing sulfonamide structures significantly influenced the inhibitory activity on these enzymes. The congruence between the findings from in vitro and in vivo studies has bolstered our understanding of the binding affinities exhibited by the most potent inhibitors at the active sites of the target enzymes. The disparities in activity and selectivity observed among the derivatives can be attributed to distinct structural variances that impart unique steric and binding properties. These findings highlight the potential of our synthesized pyrazolone derivatives as promising candidates for further research in the field of enzyme inhibition, particularly for the treatment of conditions like glaucoma and ad.

## 3 Experimental

### 3.1 General Synthetic Procedure for the Compounds

To generate a diazonium solution of sulfanilamide or metanilamide, a combination of 50 mmol of one of these substances was mixed with 15 mL of concentrated hydrochloric acid and 30 mL of water. The mixture was then cooled to 0°C–5°C. Gradually, sodium nitrite (60 mmol) dissolved in 20 mL of water was added drop by drop to the stirred mixture over a period of approximately 15–20 min. The resulting diazonium solution was stirred for about 20 min under the same low temperature conditions. This diazonium solution was subsequently combined with a separate solution of ethyl acetoacetate (obtained by dissolving 50 mmol of ethyl acetoacetate in 15 mL of methanol) while adjusting the pH to 6–7 using saturated sodium acetate. The resultant mixture was stirred at a low temperature (0°C–5°C) for 3 h and then allowed to sit overnight at room temperature in darkness. Afterward, the mixture underwent filtration, followed by several washes with cold water. The obtained material was crystallized from ethanol and used for the subsequent reaction step without any purification. In the next step, diazo‐containing ethyl acetoacetate derivatives (1 mmol) dissolved in 10 mL of ethanol were added, along with 1 mmol of substituted benzohydrazides and 4–5 drops of piperidine. The entire mixture was then refluxed for about 8–10 h with TLC (thin‐layer chromatography) monitoring. Next, the solution was filtered and washed multiple times with water. Finally, the resulting product was dried under vacuum, stored in a dark place, and thoroughly characterized using FT‐IR, ^1^H‐NMR, ^13^C‐NMR, and melting point analysis.

#### 3.1.1 4‐[(1‐Benzoyl‐3‐Methyl‐5‐Oxo‐4,5‐Dihydro‐1H‐Pyrazol‐4‐Yl)diazinyl] Benzenesulfonamide (**1a**)

Yield: 88%; Color: dark yellow solid; Melting Point: 145°C–147°C; FT‐IR (cm^−1^): 3373, 3198 (NH), 1627, 1596, 1330, 1137 (symmetric) (S=O), 1079; ^1^H‐NMR (DMSO‐d_6_, 500 MHz, δ ppm): 12.99 (s, 1H, ‐NH‐), 7.85 (d, *J* = 9.0 Hz, 2H, Ar‐H), 7.78 (d, *J* = 8.5 Hz, 2H, Ar‐H), 7.63–7.60 (m, 1H, Ar‐H), 7.50 (t, *J* = 8.0 Hz, 2H, Ar‐H), 7.38 (s, 2H, ‐SO_2_NH_2_), 2.27 (s, 3H, ‐CH_3_); ^13^C‐NMR (DMSO‐d_6_, 125 MHz, δ ppm): 165.60, 157.80, 151.60, 144.53, 140.93, 133.65, 132.75, 130.06, 128.44, 128.31, 127.71, 116.87, 12.25.

#### 3.1.2 4‐([1‐(4‐Chlorobenzoyl)‐3‐Methyl‐5‐Oxo‐4,5‐Dihydro‐1H‐Pyrazol‐4‐Yl]Diazenyl) Benzenesulfonamide (**1b**)

Yield: 88%; Color: dark yellow solid; Melting Point: 145°C–147°C; FT‐IR (cm^−1^): 3373, 3198 (NH), 1627, 1596, 1330, 1137 (symmetric) (S=O), 1079; ^1^H‐NMR (DMSO‐d_6_, 500 MHz, δ ppm): 13.12 (s, 1H, ‐NH‐), 7.94 (d, *J* = 8.5 Hz, 2H, Ar‐H), 7.83 (d, *J* = 9.0 Hz, 2H, Ar‐H), 7.66 (d, *J* = 8.5 Hz, 2H, Ar‐H), 7.61 (d, *J* = 8.5 Hz, 2H, Ar‐H), 7.35 (s, 2H, ‐SO_2_NH_2_), 2.16 (s, 3H, ‐CH_3_); ^13^C‐NMR (DMSO‐d_6_, 125 MHz, δ ppm): 165.29, 160.29, 147.50, 144.60, 140.14, 137.23, 131.62, 130.17, 129.85, 129.14, 127.79, 116.01, 12.24.

#### 3.1.3 4‐([1‐(4‐Bromobenzoyl)‐3‐Methyl‐5‐Oxo‐4,5‐Dihydro‐1H‐Pyrazol‐4‐Yl]Diazenyl) Benzenesulfonamide (**1c**)

Yield: 88%; Color: dark yellow solid; Melting Point: 145°C–147°C; FT‐IR (cm^−1^): 3373, 3198 (NH), 1627, 1596, 1330, 1137 (symmetric) (S=O), 1079; ^1^H‐NMR (DMSO‐d_6_, 500 MHz, δ ppm): 13.20 (s, 1H, ‐NH‐), 7.86 (d, *J* = 9.0 Hz, 2H, Ar‐H), 7.83 (d, *J* = 8.5 Hz, 2H, Ar‐H), 7.75 (d, *J* = 8.5 Hz, 2H, Ar‐H), 7.67 (d, *J* = 9.0 Hz, 2H, Ar‐H), 7.35 (s, 2H, ‐SO_2_NH_2_), 2.16 (s, 3H, ‐CH_3_); ^13^C‐NMR (DMSO‐d_6_, 125 MHz, δ ppm): 165.36, 160.34, 147.47, 144.55, 140.12, 132.09, 131.95, 130.23, 130.00, 127.80, 126.21, 115.97, 11.93.

#### 3.1.4 4‐([3‐Methyl‐1‐(4‐Methylbenzoyl)‐5‐Oxo‐4,5‐Dihydro‐1H‐Pyrazol‐4‐Yl]Diazenyl) Benzenesulfonamide (**1d**)

Yield: 88%; Color: dark yellow solid; Melting Point: 145°C–147°C; FT‐IR (cm^−1^): 3373, 3198 (NH), 1627, 1596, 1330, 1137 (symmetric) (S=O), 1079; ^1^H‐NMR (DMSO‐d_6_, 500 MHz, δ ppm): 13.15 (s, 1H, ‐NH‐), 7.84 (d, *J* = 4.5 Hz, 2H, Ar‐H), 7.82 (d, *J* = 4.5 Hz, 2H, Ar‐H), 7.61 (d, *J* = 9.0 Hz, 2H, Ar‐H), 7.36 (s, 2H, ‐SO_2_NH_2_), 7.32 (d, *J* = 8.0 Hz, 2H, Ar‐H), 2.37 (s, 3H, ‐CH_3_), 2.16 (s, 3H, ‐CH_3_); ^13^C‐NMR (DMSO‐d_6_, 125 MHz, δ ppm): 166.17, 160.31, 147.46, 144.49, 142.26, 140.16, 130.24, 129.46, 127.92, 115.97, 115.19, 21.48, 11.93.

#### 3.1.5 4‐([1‐(4‐Methoxybenzoyl)‐3‐Methyl‐5‐Oxo‐4,5‐Dihydro‐1H‐Pyrazol‐4‐Yl]Diazenyl) Benzenesulfonamide (**1e**)

Yield: 88%; Color: dark yellow solid; Melting Point: 145°C–147°C; FT‐IR (cm^−1^): 3373, 3198 (NH), 1627, 1596, 1330, 1137 (symmetric) (S=O), 1079; ^1^H‐NMR (DMSO‐d_6_, 500 MHz, δ ppm): 13.12 (s, 1H, ‐NH‐), 7.91 (d, *J* = 8.5 Hz, 2H, Ar‐H), 7.83 (d, *J* = 8.5 Hz, 2H, Ar‐H), 7.67 (d, *J* = 9.0 Hz, 2H, Ar‐H), 7.35 (s, 2H, ‐SO_2_NH_2_), 7.04 (d, *J* = 8.5 Hz, 2H, Ar‐H), 3.82 (s, 3H, ‐OCH_3_), 2.16 (s, 3H, ‐CH_3_); ^13^C‐NMR (DMSO‐d_6_, 125 MHz, δ ppm): 165.84, 162.43, 160.31, 147.48, 144.55, 140.12, 130.21, 129.78, 127.81, 125.21, 115.97, 114.16, 55.84, 11.93.

#### 3.1.6 4‐([1‐(3‐Chlorobenzoyl)‐3‐Methyl‐5‐Oxo‐4,5‐Dihydro‐1H‐Pyrazol‐4‐Yl]Diazenyl) Benzenesulfonamide (**1f**)

Yield: 88%; Color: dark yellow solid; Melting Point: 145°C–147°C; FT‐IR (cm^−1^): 3373, 3198 (NH), 1627, 1596, 1330, 1137 (symmetric) (S=O), 1079; ^1^H‐NMR (DMSO‐d_6_, 500 MHz, δ ppm): 13.17 (s, 1H, ‐NH‐), 7.95 (s, 1H, Ar‐H), 7.88 (d, *J* = 8.0 Hz, 2H, Ar‐H), 7.83 (d, *J* = 9.0 Hz, 2H, Ar‐H), 7.69–7.65 (m, 3H, Ar‐H), 7.57 (t, *J* = 6.8 Hz, 1H, Ar‐H), 7.34 (s, 2H, ‐SO_2_NH_2_), 2.16 (s, 3H, ‐CH_3_); ^13^C‐NMR (DMSO‐d_6_, 125 MHz, δ ppm): 164.85, 160.30, 147.47, 144.60, 140.15, 134.77, 133.87, 132.30, 131.10, 130.22, 127.71, 126.66, 115.98, 115.23, 11.93.

#### 3.1.7 3‐[(1‐Benzoyl‐3‐Methyl‐5‐Oxo‐4,5‐Dihydro‐1H‐Pyrazol‐4‐Yl)diazinyl] Benzenesulfonamide (**2a**)

Yield: 88%; Color: dark yellow solid; Melting Point: 145°C–147°C; FT‐IR (cm^−1^): 3373, 3198 (NH), 1627, 1596, 1330, 1137 (symmetric) (S=O), 1079; ^1^H‐NMR (DMSO‐d_6_, 500 MHz, δ ppm): 13.06 (s, 1H, ‐NH‐), 8.11 (s, 1H, Ar‐H), 7.84 (d, *J* = 7.0 Hz, 1H, Ar‐H), 7.78 (d, *J* = 8.5 Hz, 2H, Ar‐H), 7.64–7.57 (m, 3H, Ar‐H), 7.52–7.47 (m, 4H, Ar‐H and ‐SO_2_NH_2_), 2.27 (s, 3H, ‐CH_3_); ^13^C‐NMR (DMSO‐d_6_, 125 MHz, δ ppm): 165.59, 157.72, 151.54, 145.94, 142.52, 133.67, 132.72, 130.67, 130.00, 128.44, 127.80, 122.80, 120.34, 113.46, 12.25.

#### 3.1.8 3‐([1‐(4‐Chlorobenzoyl)‐3‐Methyl‐5‐Oxo‐4,5‐Dihydro‐1H‐Pyrazol‐4‐Yl]Diazenyl) Benzenesulfonamide (**2b**)

Yield: 88%; Color: dark yellow solid; Melting Point: 145°C–147°C; FT‐IR (cm^−1^): 3373, 3198 (NH), 1627, 1596, 1330, 1137 (symmetric) (S=O), 1079; ^1^H‐NMR (DMSO‐d_6_, 500 MHz, δ ppm): 13.05 (s, 1H, ‐NH‐), 8.11 (s, 1H, Ar‐H), 7.93 (d, *J* = 7.0 Hz, 1H, Ar‐H), 7.84 (d, *J* = 8.0 Hz, 1H, Ar‐H), 7.81 (d, *J* = 8.5 Hz, 2H, Ar‐H), 7.64–7.57 (m, 4H, Ar‐H), 7.50 (s, 2H, ‐SO_2_NH_2_), 2.27 (s, 3H, ‐CH_3_); ^13^C‐NMR (DMSO‐d_6_, 125 MHz, δ ppm): 164.49, 157.75, 151.51, 145.78, 142.39, 137.46, 132.52, 131.86, 130.76, 129.80, 128.58, 122.75, 120.37, 113.54, 12.25.

#### 3.1.9 3‐([1‐(4‐Bromobenzoyl)‐3‐Methyl‐5‐Oxo‐4,5‐Dihydro‐1H‐Pyrazol‐4‐Yl]Diazenyl) Benzenesulfonamide (**2c**)

Yield: 88%; Color: dark yellow solid; Melting Point: 145°C–147°C; FT‐IR (cm^−1^): 3373, 3198 (NH), 1627, 1596, 1330, 1137 (symmetric) (S=O), 1079; ^1^H‐NMR (DMSO‐d_6_, 500 MHz, δ ppm): 13.05 (s, 1H, ‐NH‐), 8.11 (s, 1H, Ar‐H), 7.86–7.83 (m, 2H, Ar‐H), 7.76–7.67 (m, 4H, Ar‐H), 7.65–7.58 (m, 2H, Ar‐H), 7.50 (s, 2H, ‐SO_2_NH_2_), 2.27 (s, 3H, ‐CH_3_); ^13^C‐NMR (DMSO‐d_6_, 125 MHz, δ ppm): 164.69, 157.75, 151.83, 145.89, 142.46, 132.92, 132.09, 131.51, 130.72, 130.00, 126.50, 122.86, 120.31, 113.54, 12.56.

#### 3.1.10 3‐([3‐Methyl‐1‐(4‐Methylbenzoyl)‐5‐Oxo‐4,5‐Dihydro‐1H‐Pyrazol‐4‐Yl]Diazenyl) Benzenesulfonamide (**2d**)

Yield: 88%; Color: dark yellow solid; Melting Point: 145°C–147°C; FT‐IR (cm^−1^): 3373, 3198 (NH), 1627, 1596, 1330, 1137 (symmetric) (S=O), 1079; ^1^H‐NMR (DMSO‐d_6_, 500 MHz, δ ppm): 13.00 (s, 1H, ‐NH‐), 8.11 (s, 1H, Ar‐H), 7.84 (d, *J* = 8.5 Hz, 1H, Ar‐H), 7.71 (d, *J* = 8.0 Hz, 2H, Ar‐H), 7.64–7.60 (m, 2H, Ar‐H), 7.50 (s, 2H, ‐SO_2_NH_2_), 7.31 (d, *J* = 8.0 Hz, 2H, Ar‐H), 2.39 (s, 3H, ‐CH_3_), 2.27 (s, 3H, ‐CH_3_); ^13^C‐NMR (DMSO‐d_6_, 125 MHz, δ ppm): 165.57, 157.94, 151.24, 145.95, 143.21, 142.50, 130.76, 130.36, 128.99, 127.85, 122.78, 120.30, 113.44, 21.26, 12.25.

#### 3.1.11 3‐([1‐(4‐Methoxybenzoyl)‐3‐Methyl‐5‐Oxo‐4,5‐Dihydro‐1H‐Pyrazol‐4‐Yl]Diazenyl) Benzenesulfonamide (**2e**)

Yield: 88%; Color: dark yellow solid; Melting Point: 145°C–147°C; FT‐IR (cm^−1^): 3373, 3198 (NH), 1627, 1596, 1330, 1137 (symmetric) (S=O), 1079; ^1^H‐NMR (DMSO‐d_6_, 500 MHz, δ ppm): 13.09 (s, 1H, ‐NH‐), 8.10 (s, 1H, Ar‐H), 7.83 (d, *J* = 8.5 Hz, 3H, Ar‐H), 7.64–7.60 (m, 2H, Ar‐H), 7.50 (s, 2H, ‐SO_2_NH_2_), 7.04 (d, *J* = 9.0 Hz, 2H, Ar‐H), 3.85 (s, 3H, ‐OCH_3_), 2.27 (s, 3H, ‐CH_3_); ^13^C‐NMR (DMSO‐d_6_, 125 MHz, δ ppm): 164.97, 163.25, 157.88, 150.98, 145.95, 142.55, 132.91, 130.75, 127.99, 125.30, 122.83, 120.28, 113.82, 55.89, 12.24.

#### 3.1.12 3‐([1‐(3‐Chlorobenzoyl)‐3‐Methyl‐5‐Oxo‐4,5‐Dihydro‐1H‐Pyrazol‐4‐Yl]Diazenyl) Benzenesulfonamide (**2f**)

Yield: 88%; Color: dark yellow solid; Melting Point: 145°C–147°C; FT‐IR (cm^−1^): 3373, 3198 (NH), 1627, 1596, 1330, 1137 (symmetric) (S=O), 1079; ^1^H‐NMR (DMSO‐d_6_, 500 MHz, δ ppm): 13.03 (s, 1H, ‐NH‐), 8.12 (s, 1H, Ar‐H), 7.85 (d, *J* = 8.5 Hz, 2H, Ar‐H), 7.72 (d, *J* = 7.5 Hz, 1H, Ar‐H), 7.67 (d, *J* = 7.5 Hz, 1H, Ar‐H), 7.64–7.58 (m, 2H, Ar‐H), 7.55–7.48 (m, 3H, Ar‐H and ‐SO_2_NH_2_), 7.31 (d, *J* = 8.0 Hz, 2H, Ar‐H), 2.28 (s, 3H, ‐CH_3_); ^13^C‐NMR (DMSO‐d_6_, 125 MHz, δ ppm): 164.03, 157.66, 152.15, 145.85, 142.57, 135.93, 133.08, 132.81, 130.74, 130.47, 129.49, 128.41, 127.70, 122.86, 120.39, 113.59, 12.56.

### 3.2 Biological Studies

First, purification of *h*CA I and *h*CA II enzymes for this study followed a modified procedure derived from prior literature sources [55]. To evaluate the inhibitory effects, the esterase activities of *h*CA isoforms were assessed using the p‐nitrophenylacetate substrate [56], which is converted by both isoforms to the *p*‐nitrophenolate ion, as described in our prior studies [12]. The assessment of ChE activities involved a modified adaptation of the Ellman's method [57], incorporating acetylthiocholine iodide for AChE and butyrylcholine iodide for BChE as the respective substrates. The enzymatic activity data were continuously monitored using spectrophotometry at 412 nm [58].

The inhibition effects of the novel sulfonamide‐bearing pyrazolone derivatives were determined with different inhibitor concentrations (at least five) against *h*CA isoenzymes and ChEs. The *IC*_50_ of the derivatives was calculated from Activity (%)–[inhibitor] graphs for derivatives. The inhibition types and *K*_I_ values were found by Lineweaver and Burk's curves [59]. The data analysis and graph generation were performed using GraphPad Prism version 8 for Mac (GraphPad Software, La Jolla, California, USA). The goodness of fit for enzyme inhibition models was compared using the extra sum‐of‐squares *F* test and the AICc approach. The results were presented as the mean ± standard error of the mean, along with 95% confidence intervals. Statistical significance was determined when the p‐value was less than 0.05.

### 3.3 Molecular Docking Studies

The present investigation involved utilizing the most recent version of the Small‐Molecule Drug Discovery Suite for Mac, specifically version 2023–4, to conduct molecular docking analysis [60, 61, 62]. The protein data bank (PDB) IDs, namely 1AZM [49], 3HS4 [50], 7XN1 [51], and 4BDS [52], were obtained from the RCSB Protein Data Bank. They were employed as models in the experimental setup, representing *h*CA I and II isoforms, AChE and BChE, respectively. These structures were duly prepared for docking using the Protein Preparation Wizard [63], an integral component of this suite. The molecular structures of the novel sulfonamide‐bearing pyrazolone derivatives (**1a–f** and **2a–f**) were rendered through the utilization of the ChemDraw program, specifically version 21 (PerkinElmer Inc., Waltham, MA, USA) for Mac, and subsequently optimized using the LigPrep module [64] at pH 7.4 ± 0.5 in the OPLS4 force field with Epik [65]. The active site residues authenticated by the SiteMap tool [66] were defined within the Receptor Grid Generation module [67] to generate the receptor grid within the Maestro panel [68]. The Glide application [69] used the default settings in the extra precision (XP) methodology to perform docking of the most efficacious competitive inhibitors to *h*CAs and ChEs. Additionally, the QikProp tool [70] was utilized to predict the absorption, distribution, metabolism, elimination, and toxicity (ADME/T) properties of all the targeted molecules (**1a–f** and **2a–f**) investigated in this study.

## Author Contributions

Nebih Lolak: conceptualization, formal analysis, investigation, methodology, validation, Writing, and funding acquisition. Suleyman Akocak: conceptualization, formal analysis, investigation, methodology, validation, and writing. Meryem Topal: investigation and methodology. Ümit Muhammet Koçyiğit: investigation and methodology. Mesut Işık: conceptualization and writing. Cüneyt Türkeş: conceptualization, formal analysis, investigation, methodology, validation, and writing. Fevzi Topal: investigation and methodology. Mustafa Durgun: conceptualization. Şükrü Beydemir: conceptualization.

## Conflicts of Interest

The authors declare no conflicts of interest.

## Data Availability

The data that support the findings of this study are available from the corresponding author upon reasonable request.
